# Tail biting in finishing pigs in Greek commercial farms: associations with welfare and behavioral indicators

**DOI:** 10.3389/fvets.2026.1933344

**Published:** 2026-08-18

**Authors:** Michail Kakanis, Evangelia N. Sossidou, Spyridon K. Kritas, Eleni D. Tzika

**Affiliations:** 1 Clinic of Farm Animals, Faculty of Health Sciences, School of Veterinary Medicine, Aristotle University of Thessaloniki, Thessaloniki, Greece; 2 Veterinary Research Institute, Hellenic Agricultural Organization-Demeter, Thessaloniki, Greece; 3 Department of Microbiology and Infectious Diseases, Faculty of Health Sciences, School of Veterinary Medicine, Aristotle University of Thessaloniki, Thessaloniki, Greece

**Keywords:** abattoir, behavior, bursitis, pig welfare, tail-biting, tail-docking

## Abstract

Tail biting remains a major challenge to pig welfare and the economic sustainability of pig farms, yet data from Southern European systems remain scarce. The aim of this study was to assess tail lesion prevalence, bursitis, skin lesion and behavioral risk factors for tail biting in finishing pigs, combining a slaughterhouse survey (5,765 pigs) with on-farm behavioral observation of 56 pigs. At slaughter, tail lesions were recorded in 29.4% of carcasses (27.9% mild, 1.5% severe). Intact and castrated males showed a higher severe-lesion prevalence than females, shorter tail stumps were associated with reduced severity, and lesions were more frequent in autumn, consistent with summer heat-stress exposure. Pigs with mild (28.7%) or severe (1.7%) tail lesions had a higher prevalence of hind-limb bursitis revealing a previously undescribed association (*p* = 0.007). To the best of the authors knowledge, for the first time it was documented that severe tail lesions nearly doubled (1.3%–2.2%) when posterior skin lesions were present (*p* < 0.001) suggesting that it could be used as a complementary welfare indicator at slaughterhouse. In the on-farm component, average daily gain (ADG) was a significant predictor of tail lesion severity: the odds of a higher tail lesion score rose by 73% for every 100 g/day increase in ADG (OR = 1.73, 95% CI [1.06, 2.82]). More active pigs were also more likely to direct biting behavior at penmates, and female showed more pen exploration and positive social interactions than castrated males, who spent proportionally more time in inactive states.

## 1 Introduction

Tail biting is one of the most persistent problems in intensive pig production, compromising the welfare of both biter and victim and imposing significant economic losses on producers (1) through reduced growth, carcass trimmings (2), and veterinary treatment costs. Tail-biting occurs across all husbandry systems, including outdoor production (3) and is considered a multifactorial problem resulting from the cumulative interaction of risk factors such as environmental conditions, management practices (4) and suboptimal health (5).

Four distinct types of tail-biting behavior have been described in the literature: two-stage, sudden-forceful, obsessive (6), and more recently, epidemic tail-biting (7). In the two-stage form, the behavior progresses through two phases. The first is a pre-damage stage during which a pig gently manipulates the tail of a pen mate with its mouth without causing visible lesions or a reaction from the recipient pig. The damage stage begins when the manipulation results in a skin scratch on the tail that bleeds, triggering an escalation of the behavior that can range from minor scratches to severe wounds. In the sudden-forceful type, the biter attacks another pig’s tail with no prior period of manipulation and a single biting event may result in severe tail injury such as partial tail loss or portions of skin and flesh. In the obsessive type, a pig repeatedly and forcefully, “fanatically,” bites the tails of its pen mates, often causing mild to severe injuries in several pigs in the pen. Unlike the previous type, the obsessive type is characterized by the persistent motivation of the biter to seek out and bite additional tails (6).

In the epidemic type, an abrupt environmental or management related disturbance, such as feed disruption or sudden changes in temperature, triggers mild to severe tail biting in one or more pens that spreads rapidly throughout (7).

The majority of European pig farmers dock pig’s tails early in life (8), a practice maintained primarily because tail-biting episodes are sporadic, unpredictable, and prone to rapid escalation into outbreaks (9). Nevertheless, routine tail docking is prohibited under EU legislation (10) and the European Commission has issued specific management recommendations (11) to reduce tail biting risk and the consequent reliance on docking.

Accurate data regarding the proportion of pigs subjected to tail docking across different EU member states is lacking and there is an even greater scarcity of information concerning the prevalence of tail biting within these populations (12). The available literature is also geographically skewed as most published studies originate from Northern European production systems (13–15), while Southern European systems, characterized by different farm infrastructure, ventilation strategies and climatic exposure, remain largely undocumented. In Greece, to date, the only available data derive from a survey conducted within the framework of GroupHouseNet, COST Action 15,134, which reported tail-biting lesions in a small number of animals confirming that the issue is relevant to the Greek context (16). However, no systematic investigation has since been undertaken.

EU legislation does not specify the length of the tail that should be removed when docking is performed, yet the consequences of different docking lengths for subsequent tail-biting risk have received comparatively little research attention. Scollo et al. (17) in an epidemiological study reported a lower prevalence of tail lesions in pigs docked to less than half their original tail length compared with pigs docked more conservatively while Thodberg et al. (9) similarly found docking length to modulate lesion severity. Earlier studies reporting no clear benefit of tail docking (18) involving different production conditions and scoring scales, and therefore complicating any direct comparison.

Despite widespread recognition that climatic conditions influence pig health and welfare, longitudinal studies on this relationship remain scarce (19), and the existing evidence on temperature effects on tail biting is contradictory. Some experimental (20) and epidemiological studies (19, 21) report higher tail-biting prevalence at low ambient temperatures, while others document increased tail biting under high temperature conditions (22) particularly when ventilation systems are compromised (23). This discrepancy may reflect genuine differences in the influence of climatic conditions on the production systems studied, with cold-stress effecting predominantly Northern European systems and heat-stress emerging in Mediterranean systems: a hypothesis that remains undertested in the Southern European context.

Bursitis is a lesion most commonly observed on the metatarsal region of the hind limbs of pigs (24). There are fluid-filled sacs that form in the subcutaneous connective tissue in response to prolonged mechanical pressure exerted on the skin particularly over bony prominences (25). Initially, the sacs contain serous fluid, with granulomatous tissue developing at later stages. Bursitis formation is primarily associated with repeated trauma to the affected region (26, 27) with type of flooring and absence of bedding identified as key risk factors (28). Bursitis prevalence therefore reflects suboptimal environmental conditions on farms (24, 29) and is associated with lameness, a recognized determinant of compromised pig welfare across all of the Five Freedoms (30).

Skin lesions have been identified as significant indicators of pig welfare due to the ease with which they can be recorded and used to identify welfare problems at farm level (31, 32). Veit et al. (33) suggest that skin lesions may result from increased agonistic behavior that develops after mixing unfamiliar pigs. Furthermore, skin lesions acquired in stable hierarchical groups, well after the grouping process, likely from ongoing competition over resources (34), remain detectable at slaughterhouse up to 10 weeks after formation (35). Whether the anatomical distribution of skin lesions corresponds to tail-biting victim status, however, has received little empirical attention.

The present study had three integrated aims: (i) to provide the first systematic estimate of the prevalence of tail biting lesions, hind limb bursitis and skin lesions in slaughter pigs in Greece and to examine their seasonal patterns, (ii) to evaluate whether tail stump length modulates tail biting lesion severity in commercially docked populations, and (iii) to investigate the relationship between individual behaviors, growth performance and tail lesion severity in finishing pigs through on farm behavioral observations.

## 2 Materials and methods

### 2.1 Study 1

The minimum required sample size was calculated using the statistical software G*Power 3.1.9. The analysis was run using *z*-test family and proportions: difference between two independent proportions test, with desired Power 0.8 and type I error 0.05. The parameter of interest was the prevalence of tail biting lesions between males, castrated males and females. A literature review found that the average prevalence of tail biting lesions recorded in abattoir studies was approximately 30% (13, 36, 37). Furthermore, the difference between the prevalence in intact males and females was found to be more than 6%. Accordingly, the desired detectable difference of the parameter of interest was set at 6%. Considering that females and castrated males represent approximately 50 and 40% of the animals slaughtered in abattoirs in Greece, the ratio of intact males to females was established at 0.2. The analysis revealed that, at least, 2,523 females and 504 intact males were required (actual Power 0.800). The same analysis between females and castrated males demonstrated that, at least, 963 females and 770 castrated males (actual Power 0.800) were required. To achieve these goals, 5,765 animals (2,789 females, 616 intact males and 2,360 castrated males) were evaluated for the purpose of the study.

This study was conducted at a pig slaughterhouse in the north of Greece over for a period of 16 months of non-consecutive days, between July 2018 and October 2019, covering all four seasons. On each study day, all pigs in the batches arriving were included in the analysis. Carcasses were inspected on the slaughter line immediately after evisceration and it involved finishing pigs (between 150–170 days of age, and a mean slaughter weight of approximately 110 kg). Pig sex, herd of origin and tail, skin and hind limb bursitis scoring were recorded by a single trained veterinarian throughout the study period to ensure scoring consistency. Tail lesions severity was scored according to a 5-point scale (38):

- 0 No evidence of tail biting,
- 1 Healed or mild lesions,
- 2 Evidence of chewing or puncture wounds, but no evidence of swelling,
- 3 Evidence of chewing or puncture wounds with swelling and signs of possible infection,
- 4 Partial or total loss of the tail.

Remaining tail length was visually categorized as being either short (≤10 cm) or long (>10 cm) with the cut-off chosen to reflect the most common docking practice in Greek farms. Due to low prevalence of many of the scores, results were summed in 0–1-2 scores for analysis. Score 0 indicates healthy tail (original score 0), score 1 minor lesions (original scores 1–2) and score 2 tails with severe lesions (original scores 3–4). Hind-limb bursitis was recorded using an adapted scale of Harley et al. (39) as present (1) or absent (0).

Three major areas of the body were assessed for skin lesions, namely; head, shoulders and front legs (Front area), left and right flank (Middle area) and hindlegs and tail area (Posterior). An adapted six point scoring system (35) was used where only healed lesions (non-red) were recorded (Table 1). However, due to the high prevalence of no lesions we aggregated the data into a binary variable were 0 = no lesions and 1 = presence of skin lesions.

**Table 1:** Skin lesion scoring method for pigs.

Score	Description
0	No injuries
1	One small (approx. 2 cm) superficial lesion (not penetrating the skin)
2	More than one small, superficial lesion
3	One or several big (2–5 cm) and deep (a lesion penetrating the skin) lesions. If deep, only one single lesion
4	One very big (>5 cm), deep lesion
5	Many very big, deep lesions covering the skin area

### 2.2 Study 2

The second part of the investigation took place in a 350 sows farrow to finish farm in the north of Greece from April to July 2021. A total of 56 growing-finishing pigs [(Landrace × Large White) × Duroc], at the age of 8 weeks were housed in eight structurally identical pens. Manipulable material according to normal farm practices were used to all pens. Pigs were tail-docked, male pigs castrated at the age of 3 days while teeth clipping was not performed. The dimensions of each pen were 2.20 m × 2.65 m with partially slated floor and was equipped with a nipple drinker and a single-space round feeder so that the animals had *ad libitum* access to water and a standard finisher diet (space allowance per pig was according to the standards of European Union legislation). Pigs were weighed at the start of the investigation and at the end of the finishing period (approximately, at the age of 22 weeks) and the average daily gain (ADG) was calculated as the last weight minus the first weight measured for every pig divided by the number of days between the weight measurement. Tails were scored for lesions at the end of the finishing period based on the scoring system mentioned above.

In scientific literature, behavioral observational studies are predominantly conducted in experimental facilities due to the practical challenges of data collection in commercial farms. Valros et al. (40), who performed behavioral observations across eight commercial farms during the growing and finishing period, described an observational protocol. Based on their framework and taking into consideration the technical limitations existing in the investigation farm, the following observational protocol was applied in the present study. Behavioral observations were made through the use of video cameras utilizing instantaneous scan sampling at 20-min intervals between 09:00 and 17:00 once a week for 10 consecutive weeks resulting in 24 scans per pig per day. Four security cameras (Hikvision DS-2CD2051G1-IDW, 2.8 mm, Hikvision Digital Technology Co, Ltd.) were installed above the pens at a height of approximately 5 m above the ground, with each camera recording two pens. Pigs were individually identified by an ear tag with a number code and were marked by a unique symbol using a non-toxic spray in the morning of the observation day. Any periods when caretakers entered the room were excluded from the analysis.

Behaviors were quantified as relative frequencies using a detailed ethogram consisting of 26 distinct behavioral observations. The ethogram utilized in the present study was established through an extensive literature review. It was mainly adapted from the scientific work of Valros et al. (40), while also took under consideration ethograms described in other behavioral studies (41, 42). Additionally, for the final ethogram creation, the observations of tail biting behavior that were collected through a 4-week pilot study were also taken into consideration. Moreover, in order to have a detailed ethogram, manipulative behaviors directed at pen mates and exploration of pen fixtures were recorded in all three positions of the body either in sternal recumbency, in dog position or standing on all four legs. Due to the fact that some manipulation behaviors directed at pen mates or pen fixtures were too few for a lot of pigs we aggregated those in new variables according to Table 2.

**Table 2:** Detailed ethogram of assessed behaviors of pigs.

Behavior	Description	Activity	Social
Mounting	Standing on hind legs while having front legs on other pigs back	Active	Negative
Locomotion	Walking/running/standing on all four legs, without performing any other behavior	Active	Negative
Tail biting	Nosing, sniffing, touching or biting the tail of penmate with the snout	Active	Biting
Ear biting	Nosing, sniffing, touching or biting the ear of penmate with the snout	Active	Biting
Other biting	Touching, sniffing, rooting, chewing, licking or sucking part of the body of a pen-mate, causing a reaction of the other pig	Active	Negative
Lateral/sternal recumbency	Lying without performing any other behavior	Inactive	Neutral
Exploring other pigs	Touching, sniffing, rooting, chewing, licking, nibbling or sucking part of the body of a pen-mate, without causing a reaction in the other pig	Active	Positive
Sitting on a “dog position”	Sitting on the tail with the forelegs stretched straight under the body without doing any of the other below listed activities	Active	Neutral
Exploring the floor	Exploring the floor or substrate on the floor by sniffing, nosing, rubbing, licking, rooting or chewing it.	Active	Exploration
Exploring the pen	Exploring any part of the pen except the floor by nosing, sniffing, touching, licking, chewing or sucking	Active	Exploration
Eating/drinking	Eating in the feeder of drinking water from the nipple	Active	Neutral

All experimental procedures, animal handling, and data collection protocols were reviewed and approved by the Directorate of Agricultural and Veterinary Services of Central Macedonia Region and by the bioethics committee of the School of Veterinary Medicine, Faculty of Health Sciences, Aristotle University of Thessaloniki.

### 2.3 Data management and analysis

PS IMAGO PRO 10 (SPSS statistics 29) was used for all statistical analyses of this study.

In study 1 chi-square test was used to assess the differences of the percentages of the parameters evaluated between tail lesions scores set.

In study 2 assessment of normality of social behavior variables was based on the Shapiro–Wilk test and visual inspection of histograms and Q–Q plots. Evaluation of the bivariate relationships between overall activity, specific negative behaviors, growth rates and tail lesion scores were calculated using Spearman’s Rank Order Correlation. Differences between the three tail lesion severity groups in their behaviors was evaluated using Kruskal–Wallis *H* test. To investigate the relationship between overall activity and the behaviors in the bivariate analysis we used the Spearman’s Rank-Order Correlation.

To estimate the likelihood of an individual pig having a more severe tail lesion score we used an Ordinal Logistic Regression model which included growth rate, active behavior, biting behavior and gender as factors. To generate Odds Ratios (OR) that are biologically meaningful, continuous predictors were mathematically scaled prior to modeling: relative behavioral frequencies were multiplied by 10 (explainable as a 10% increase in the total time budget), and ADG (kg/day) was multiplied by 10 (explainable as a 100 g/day increase in growth). The model made use of a Logit link function, and full parameter estimates, including 95% confidence intervals (CI), were exponentiated to report final Odds Ratios.

All comparisons were made at significance levels of *α* = 0.05.

## 3 Results

### 3.1 Study 1

Overall, 5,765 pigs were assessed, comprising 48.4% females, 40.9% castrated males, and 10.7% intact males, as presented in Table 3. Tail lesions were observed in 29.4% of pigs, while severe tail lesions were recorded in 1.5%. The distribution of the tail biting lesions is presented in Table 4.

**Table 3:** Number and percentage of female, male, and castrated male pigs assessed at slaughterhouse.

Gender	Pigs	Percentage (%)
Female	2,789	48.4
Male	616	10.7
Castrate	2,360	40.9
Total	5,765	100%

**Table 4:** Distribution of tail biting lesions (number and percentage of pigs per category).

Tail lesion scores	Pigs	Percentage (%)
Score 0	4,068	70.6
Score 1	1,256	21.8
Score 2	354	6.1
Score 3	52	0.9
Score 4	35	0.6

As indicated in Table 5, pigs with longer tails showed significant differences (*p* < 0.05) across all lesion categories compared with pigs with shorter tails.

**Table 5:** Distribution of tail lesions in pigs by tail length category and lesion severity (percentage % and absolute number).

Tail lesion scores	Tail length<10 cm	Tail length>10 cm
No lesions (0)	73.9^a^ (2932)	63.1^b^ (1136)
Mild (1, 2)	25.9^a^ (1029)	32.3^b^ (581)
Severe (3, 4)	0.1^a^ (5)	4.6^b^ (82)

a, b: Different superscripts in the same row indicate statistically significant differences (*p* < 0.05).

A greater proportion of intact and castrated males exhibited severe lesions compared to females, and the difference was significant (*p* < 0.05; Table 6).

**Table 6:** Distribution of tail lesions in pigs by sex (percentage % and absolute number).

Tail lesion scores	Castrates	Males	Females
No lesions (0)	70.8^a^ (1671)	59.7^b^ (368)	72.8^a^ (2029)
Mild (1, 2)	27.3^a^ (644)	37.3^b^ (230)	26.4^a^ (736)
Severe (3, 4)	1.9^a^ (45)	2.9^a^ (18)	0.9^b^ (24)

a, b: Different superscripts in the same row indicate statistically significant differences (*p* < 0.05).

A significantly higher percentage in hind limb bursitis was observed in pigs with severe tail lesions (1.7%) and mild tail lesions (28.7%) compared with pigs without tail lesions (Table 7) while pigs with severe tail lesions also exhibited a significantly higher percentage in skin lesions on the caudal region of the body (Table 8).

**Table 7:** Distribution of tail lesions in pigs by hind limb bursitis and lesion severity (percentage% and absolute number).

Tail lesion scores	Hind limb bursitis score	
	No lesion	Lesion
Mild	25.5^a^ (332)	28.7^b^ (1274)
Severe	0.9^a^ (12)	1.7^b^ (75)

a, b: Different superscripts in the same row indicate statistically significant differences (*p* < 0.05).

**Table 8:** Distribution of tail lesions in pigs by skin lesion severity (percentage % and absolute number).

Tail lesion score	Skin lesion score by body part					
	Front part		Middle part		Hind part	
	No lesion	Lesion	No lesion	Lesion	No lesion	Lesion
Mild	26.2^a^	34.7^b^	25.7^a^	34.2^b^	25.7^a^	36.4^b^
	(1167)	(439)	(1068)	(539)	(1148)	(459)
Severe	1.5^a^	1.7^a^	1.5^a^	1.6^a^	1.3^a^	2.2^b^
	(66)	(21)	(62)	(25)	(59)	(28)

a, b: Different superscripts in the same row indicate statistically significant differences (*p* < 0.05).

A progressively increasing trend in tail lesion prevalence was observed across seasons, particularly from winter to autumn. While 62.7% of pigs showed no lesions during autumn, this proportion increased to 76.4% in winter. The data indicate a significant difference in the occurrence of mild tail lesions between autumn and all other seasons, as well as between summer and winter. Although the number of severe lesion cases was low, a significant difference was observed in autumn and summer compared to winter (Table 9).

**Table 9:** Distribution of tail lesions in pigs by season and lesion severity (percentage % and absolute number).

Tail lesion scores	Winter	Spring	Summer	Autumn
No lesions (0)	76.40^a^ (735)	74.01^a^ (1028)	69.28^b^ (1730)	62.70^c^ (725)
Mild (1, 2)	22.97^a^ (221)	25.05^ab^ (348)	28.87^a^ (721)	34.90^c^ (320)
Severe (3, 4)	0.62^a^ (6)	0.94^ab^ (13)	1.84b^c^ (46)	2.40^c^ (22)

a, b, c: Different superscripts in the same row indicate statistically significant differences (*p* < 0.05).

### 3.2 Study 2

The average weight at the start of the experiment was 25.3 (±3.5) kg and at the end of the finishing period 100.2 (±12.72) kg. ADG during this period was on average 0.727 (±0.111). Twenty-nine pigs were castrated males and 27 females. Out of 56 pigs observed in this study, 27 (48.2%) had a minor tail lesion, 12 (21.4%) had a severe lesion while 17 (30.4%) had no lesions at all. Lateral and sternal recumbency together accounted for almost 71% of all observations. The bivariate correlation analysis showed that the proportion of activity was not significantly associated with ADG (*ρ* = −0.088, *p* = 0.521). Biting behaviors were statistically significantly correlated with overall activity levels (*ρ* = 0.297, *p* = 0.026). Additionally, a significant positive relationship between ADG and tail lesion severity score (*ρ* = 0.285, *p* = 0.034) was found.

Female pigs engaged in significantly higher levels of positive social behaviors toward their penmates compared to males (median [IQR]:2.39% [1.43–3.82%] vs. 1.91% [0.95–1.91%]) respectively (*p* < 0.05) and pen exploration (*p* < 0.05). Conversely, male pigs allocated a significantly larger proportion of their time to socially neutral behaviors (*p* = 0.04). Notably, biting behaviors did not significantly differ between sexes (*p* > 0.05). Descriptive analysis of performance metrics revealed a positive correlation between growth and tail lesion severity. Specifically pigs with tail lesion score 0 had the lowest average daily growth at 0.680 (0.126) while pigs with tail lesion score 1 and 2 recorded higher 0.742 (0.098) and the highest 0.761 (0.102) average growth, respectively. The Ordinal Regression model revealed significant association between ADG and the severity of the tail lesion (*p* = 0.028). Specifically, for every 100 g/day increase in a pig’s average daily growth, the odds of that individual to have a more severe tail lesion increased by 73% (OR = 1.73, 95% CI [1.06, 2.82]). Furthermore, the model demonstrated that neither a pig’s overall activity level (*p* = 0.765) nor its own engagement in biting behaviors (*p* > 0.05) significantly predicted its lesion severity.

## 4 Discussion

This study provides the first systematic assessment of tail-biting lesion prevalence and behavioral expression in intensively reared pigs in Greece—a region under presented in EU pig-welfare data. Although slaughterhouse-based welfare monitoring is gaining traction across the European Union, comparable data from Southern Europe remain scarce despite marked differences in farm infrastructure and climate compared with Central and Northern Europe. In the present study, 29.4% of the evaluated slaughter pigs exhibited tail biting lesions (TBL), of which 27.9% were mild and 1.5% severe (STL). The prevalence of severe tail lesions observed in this study is consistent with the range reported in other studies (43, 44). In addition, Franco et al. (45) in a study conducted in Portugal reported an overall tail lesion prevalence of approximately 15%, with severe tail lesions accounting for 2.1% of the cases, a prevalence comparable to that observed in the present study. Likewise, a study involving weaner pigs in the same country documented an overall tail lesion prevalence of 21.5% (46) Furthermore, a recent study from Chile, where pig production systems and climatic conditions differ from those typically encountered in Europe, reported a prevalence of severe tail lesions of up to 4.28% (47).

However, the total prevalence of tail lesions (29.4%) was lower than those reported by Harley et al. (48) (~58%), Harley et al. (39) (~72%) Teixeira et al. (15) (~72%) and Vitali et al. (~35%) (49). Lower prevalence of tail lesions observed in the present research may be attributed to the high proportion of pigs with docked tails. All pigs evaluated in the study came from farms practicing tail docking, and ~70% had been docked to short remnants. Furthermore, comparison with past studies is considered a challenging task as data collection includes differences in docking status, a variety of scoring systems and age at evaluation. For example, Vitali et al. (49) reported a prevalence of 34.8%. However, their study population consisted of pigs slaughtered at a live weight of 160 kg ± 10% and at least 9 months of age, whereas pigs in Greece are typically slaughtered at approximately 5.5 months of age.

Furthermore, country-specific variation in on-farm management and housing conditions may influence prevalence and severity of TBL (50) while several studies reporting higher TBL prevalence were conducted in countries with strict legislation against routine docking and therefore involved undocked pigs (51, 52). Moreover, different points of observation on the slaughterhouse line can itself influence recorded prevalence (36) as also the scope of recording, i.e., routine meat inspection vs. welfare research (44).

In this study, approximately 70% of the pigs examined had undergone tail docking, removed more than two-thirds of the original tail length, leaving one-third or less intact. This is broadly consistent with a previous national survey reporting that 55.6% of pig farmers in Greece retain one-third or less of the tail (53), although the proportion observed here was somewhat higher. A statistically significant association was observed between tail-stump length and the severity of tail biting lesions (*p* < 0.05). These results align with previous evidence that tail docking reduces TBL prevalence in growing-finishing pigs (54, 55) and that docking length further modulates lesion severity (9). A possible explanation for the reduced severity of tail lesions in pigs with short docked tails could be that “biter” pigs find it difficult to grasp a short tail (56) which may go unnoticed or be less attractive due to its reduced size (57, 58).

A statistically significant sex effect was also detected: intact and castrated males showed higher severe TBL prevalence than females (*p* < 0.05), consistent with previous reports (2, 38, 59). Although no single mechanism has been firmly established, female pigs approaching puberty and sexual maturity exhibit elevated activity (58) and increased manipulation of penmates’ tails (13) which may translate into more biting directed at male and castrated penmates. Another hypothesis is that castrated males may be less reactive to tail manipulation, as they tend to be heavier and more docile (60, 61) and therefore, by occupying feeding spaces more frequently, they become targets of aggressive behavior from female pigs (62, 63).

This study also offers an initial estimate of bursitis prevalence in Greek slaughter pigs, contributing at the same time to the enrichment of the available European data. Bursitis of the hind limbs was observed in slightly more than three quarters of the evaluated population (77.3%). The prevalence of bursitis shows a wide range across different studies: in the UK it has been reported at 87% in Scotland (26); and between 41.2 and 63.4% in England (24, 64); in Ireland the prevalence only for severe lesions was 44% (39). Furthermore in a study of heavy pigs reared in the Parma region of Italy, the rate was 24.7% (65) while in a more recent study in German pig fattening units the prevalence of bursa auxiliaries was 76% (66). The prevalence of bursitis observed in Greece may be attributed to different rearing conditions compared to other countries. Greek farms predominantly use slatted concrete floors, especially in finishing pens, and straw bedding is not utilized often due to the challenges it poses for the operation of slurry removal systems (67) and the risk of heat stress in the context of warmer weather. Both factors have been linked to bursitis occurrence (24, 26, 64). A notable finding of the present study is the strong, positive association between tail lesions of any severity with bursitis. While bursitis prevalence has been documented in other epidemiological studies, an association of this kind has not, to the best of the authors knowledge, been described previously. This finding may partly reflect the prolonged housing duration of pigs with tail lesions, which take longer to reach slaughter weight in farms with high rates of severe TBL (1). A possible explanation could be that pigs with severe tail lesions, in an attempt to protect their tail from biters, frequently adopt a “sitting dog” posture when pen space is insufficient to escape biters; this posture better protects the bitten tail but concentrates body weight on the hind limbs that has been linked to increased bursitis risk (64), thereby increasing the risk of bursitis. Moreover, as pigs spend most of their time lying down (68) prolonged contact with slatted flooring is expected, leading to an increased risk of bursitis.

The present study examined the relationship between skin lesions and tail biting, an association that, to the authors knowledge, has not previously been investigated, even though slaughterhouse skin lesion scoring can reflect on-farm welfare up to 10 weeks prior to slaughter (35). Results of this study indicated a positive correlation between the presence of skin lesions on the posterior region of the body and severe TBL. A possible explanation could be that victim pigs are subject to more aggressive behavior on the posterior region of their body compared to other pigs, as well as the fact that victims typically receive repeated bites in the tail region (69, 70). At the phenotypic level, Turner et al. (71) suggest that skin lesions on the anterior region of the body are primarily associated with mutual aggressive interactions between two pigs, whereas posterior lesions are mainly the result of unilateral aggressive actions by other pigs that are not reciprocated by the victim; a pattern consistent with the tendency of victim pigs not to retaliate but simply escape the aggressor pig. An additional contributor may be competition to gain access to the feeding area, which often results in bites on the posterior region of the pigs already at the trough. This aggressive behavior can be a result of stress (72) and can be attributed to the second form of tail biting behavior, namely, the “sudden-forceful” (6). Furthermore, research by Desire et al. (73) indicates that pigs with a high number of skin lesions living in hierarchically stable groups are the least aggressive animals, which supports the results of this study.

The present study collected data on tail biting lesions for over a year, allowing the recording of lesions across all four seasons. A seasonal variation in the prevalence of lesions was observed, specifically a significant trend toward an increase in autumn compared to winter. Tail biting episodes typically occur after weaning (74, 75). Therefore, considering the slaughter age of the pigs in this study, it can be hypothesized that the cases recorded in autumn at the slaughterhouse were due to tail biting incidents that occurred on the farm during the summer months. Greece experiences its highest temperatures during this period of year and most of the farms employ natural ventilation systems supplemented by fans. Since the capacity of ventilation and cooling systems in pig housing is not unlimited (67), indoor temperatures could exceed pig’s thermoneutral zone. Heat stress has been identified as a significant factor in the occurrence of tail biting behavior (6, 58). The present’s study findings align with experimental work by Holling et al. (23), who recorded increased tail-biting rates when very high temperatures prevailed in the chambers due to ventilation failure, and with previous work by Geers et al. (76) who argue that inadequate maintenance of ideal temperatures in the housing is a potential cause of tail biting. Italian slaughterhouse data similarly show higher TBL prevalence in the warmer period of the year (22).

However, these findings contrast with epidemiological data from Northern Europe. where fewer cases are reported in summer and autumn (19, 21). Summer temperatures in those regions rarely exceed 30 °C, so heat stress possibly does not affect the animals to the same extent. This notion is supported by the conclusions of Bottacini et al. (77), who recorded a lower TBL prevalence in winter compared to other seasons in Italy, attributing this pattern to higher average ambient temperatures in Italy than the other countries.

The on-farm observational study revealed a significant positive association between average daily gain (ADG) in the finishing period and tail lesion severity. Pigs growing faster had a greater chance of having a higher tail lesion score than their penmates. Specifically, for every 100 g/day increase in a pig’s average daily gain the odds of higher TLS was raised by 73%. It is often hypothesized that heavier pigs are staying at the feeder more frequently, exposing their tails to other pigs in the pen for longer (6, 62). These results are consistent with Palander et al. (78) who suggested that reducing the visits to the feeder could be a strategy to avoid being bitten. It is plausible, the fastest growing pigs in the present study had the highest nutritional demand and therefore the strongest motivation to feed, leading to longer feeder occupancy and consequently more tail-directed interactions from penmates.

Consistent with this, Zonderland et al. (79) showed that heavier pigs at weaning are more likely to be victims and Hakansson and Houe (63) reported that a higher growth rate after weaning is considered to raise the risk for tail lesions. Conversely, Camerlink et al. (41) and Sinisalo et al. (80) reported reduced weight gain in pigs that received more tail bites during finishing. This discrepancy likely reflects different victim definitions as the latter study included severely bitten pigs which were essentially absent in the present sample. The feeder area is generally considered a high-risk zone for tail biting (78).

Pigs that spend more time active were also more likely to engage in biting behavior toward other pigs. This is consistent with prior reports that tail biters direct their exploratory behavior at penmates (79) or enrichment materials (70). Moreover, these results agree with earlier research that found higher activity levels of pigs in pens with higher tail lesions (42, 81, 82) who showed that ongoing tail biting behavior, even if it is not resulting in bleeding tail lesions as in this study, result in changes in pen activity. Individual level activity monitoring may therefore provide a tractable indicator for identifying biters within a pen; a finding with potential applications for early detection based in precision livestock farming tools.

It was further hypothesized that tail lesion status would be reflected in pigs’ social behavior. Contrary to this expectation, having tail lesions were not associated with any of the social behaviors examined (Table 2). This partly contrasts with Hakansson et al. (83), who reported differences between victims and biters in two behaviors, suckling and mounting; however, their work was conducted on preweaning piglets whereas the evaluated pigs were in the finishing phase.

Notably, tail biting in this study never escalated to a ‘tail biting outbreak’ as mostly described in the literature. Biting frequency fluctuated narrowly between 0.4 and 1.6% of observations across all days, with no temporal trend. The inability to detect an association between general behavioral patterns and tail lesion scores may stem from the overall absence of severe lesions; such a low prevalence inherently restricts the capacity to discern strong correlations.

Castrated males and female also differed significantly on some social behaviors. Specifically females showed more pen exploration which is in line with Paoli et al. (56) who reported that female weaned pigs perform more investigatory behaviors such as enrichment use and Breuer et al. (84) where female weaned pigs in a “Tail Chew Test” showed a tendency to manipulate the rope more frequently than intact males. Furthermore, female pigs engaged more in positive interactions with penmates than male pigs, whereas males spent a significantly higher proportion of their time in neutral states. Zonderland et al. (85) reported that females were more likely to be biters than intact males. It has been suggested that differences between sexes may be observed because males are typically considered bigger and less active than females, which possibly makes them predisposed to tail biting by penmates (61).

## 5 Conclusion

To the best of the authors knowledge, this is the first study to evaluate the prevalence of tail biting lesions in finishers pigs slaughtered in Greece. Despite the limitations of the present study, specifically the low on farm prevalence of severe tail lesions and limited weekly frequency of behavioral observations, although these observations were performed for a prolonged period of time, it was confirmed that tail lesions are common in Greek slaughter pigs while the prevalence of mild and severe tail lesions illustrate that there is an ongoing tail biting problem in pig farms of the country, despite the high percentages of tail docking.

Under Southeastern Europe climatic conditions, not previously examined in this respect, a correlation between season of the year and tail lesion severity was also revealed, however further studies are needed to confirm this relationship. In addition to reporting the prevalence of bursitis in intensive pig farming in Greece, the present study showed a positive association between tail lesions of any severity with hind limb bursitis suggesting that it could be used as a complementary welfare indicator at slaughterhouse. An association between tail biting lesions and skin lesions is also reported for the first time, indicating that tail biting lesions severity could be used to assess broader welfare outcomes of the farm.

These findings suggest that farmers could reduce the risk of tail biting by

- Improving thermal management during the hot season to mitigate heat-stress as a risk factor for tail-biting.
- Incorporating fast-growing males and individual activity into their tail biting risk assessment protocols.

## Data Availability

The data supporting the findings of this study are not publicly available due to GDPR restrictions relating to personal data of the participating farmers and commercial sensitive information. Requests to access the datasets should be directed to mkakanis@vet.auth.gr.
